# Effect of prewarming on body temperature in short-term bladder or prostatic transurethral resection under general anesthesia: A randomized, double-blind, controlled trial

**DOI:** 10.1038/s41598-021-00350-2

**Published:** 2021-10-21

**Authors:** Ángel Becerra, Lucía Valencia, Pedro Saavedra, Aurelio Rodríguez-Pérez, Jesús Villar

**Affiliations:** 1 Department of Anesthesiology, Hospital Universitario de Gran Canaria Dr. Negrín, Las Palmas de Gran Canaria, Spain; 2 Department of Medical and Surgical Sciences, University of Las Palmas de Gran Canaria, Las Palmas de Gran Canaria, Spain; 3 Department of Mathematics, University of Las Palmas de Gran Canaria, Las Palmas de Gran Canaria, Spain; 4 CIBER de Enfermedades Respiratorias, Instituto de Salud Carlos III, Madrid, Spain; 5 Research Unit, Hospital Universitario de Gran Canaria Dr. Negrín, Las Palmas de Gran Canaria, Spain; 6 Keenan Research Center for Biomedical Sciences at the Li-Ka Shing Knowledge Institute, St. Michael’s Hospital, Toronto, ON Canada

**Keywords:** Outcomes research, Risk factors, Comorbidities, Pain

## Abstract

Perioperative hypothermia causes postoperative complications. Prewarming reduces body temperature decrease in long-term surgeries. We aimed to assess the effect of different time-periods of prewarming on perioperative temperature in short-term transurethral resection under general anesthesia. Randomized, double-blind, controlled trial in patients scheduled for bladder or prostatic transurethral resection under general anesthesia. Eligible patients were randomly assigned to receive no-prewarming or prewarming during 15, 30, or 45 min using a forced-air blanket in the pre-anesthesia period. Tympanic temperature was used prior to induction of anesthesia and esophageal temperature intraoperatively. Primary outcome was the difference in core temperature among groups from the induction of general anesthesia until the end of surgery. Repeated measures multivariate analysis of covariance modeled the temperature response at each observation time according to prewarming. We examined modeled contrasts between temperature variables in subjects according to prophylaxis. We enrolled 297 patients and randomly assigned 76 patients to control group, 74 patients to 15-min group, 73 patients to 30-min group, and 74 patients to the 45-min group. Temperature in the control group before induction was 36.5 ± 0.5 °C. After prewarming, core temperature was significantly higher in 15- and 30-min groups (36.8 ± 0.5 °C, p = 0.004; 36.7 ± 0.5 °C, p = 0.041, respectively). Body temperature at the end of surgery was significantly lower in the control group (35.8 ± 0.6 °C) than in the three prewarmed groups (36.3 ± 0.6 °C in 15-min, 36.3 ± 0.5 °C in 30-min, and 36.3 ± 0.6 °C in 45-min group) (p < 0.001). Prewarming prior to short-term transurethral resection under general anesthesia reduced the body temperature drop during the perioperative period. These time-periods of prewarming also reduced the rate of postoperative complications.

*Study Registration* Registered at ClinicalTrials.gov (Identifier: NCT03630887).

## Introduction

Perioperative hypothermia is common in patients under general anesthesia^1^. Its appearance could lead to cardiovascular and hemorrhagic complications^1,2^. Since duration of surgery is one of the main risk factors for intraoperative temperature decrease^3^, most reported studies dealing with anesthesia-associated hypothermia have focused on long-term surgical procedures. However, the decrease in body temperature starts just after anesthesia induction due to impaired thermal regulation, loss of compensatory mechanisms by anesthetic-induced vasodilation, and redistribution of heat from core to peripheral compartments. Patients are usually normothermic at the end of surgery when intraoperative active warming is applied. However, patients undergoing short-term surgeries are usually hypothermic at the end of surgery, even with intraoperative warming, due to redistribution hypothermia^4^.

During the first 30 min after anesthetic induction, an abrupt decrease in temperature from 0.5 to 1.5 °C occurs^4^. One of the most effective tools for preventing perioperative hypothermia is the application of active prewarming using forced-air devices. Most recent guidelines recommend its application before anesthetic induction^5–7^. However, it is surprising that this recommendation applies only to long surgeries^6^, since the fastest temperature decrease occurs just after anesthesia induction^8^. There are no prewarming recommendations for short-term surgical procedures due to lack of evidence.

We hypothesized that short-time periods of preoperative forced-air warming (for 15, 30 or 45 min) will prevent the decrease of body temperature in patients undergoing transurethral resection under general anesthesia, when compared to non-prewarmed patients. As secondary objectives, we assessed the prevalence of hypothermia among different groups upon arrival at Post-Anesthesia Care Unit (PACU) and examined the effects of these prewarming times for decreasing the rate of postoperative shivering, postoperative pain, length of stay in the PACU, and development of other postoperative complications.

## Materials and methods

### Study design and patients

This study was approved by the Hospital’s Institutional Review Board (CEI/CEIm Provincial Las Palmas, approval number 2018-153-1). Written informed consent was obtained from all subjects participating in the trial. The trial was registered prior to patient enrollment at clinicaltrials.gov (NCT03630887) on 15/08/2018. This manuscript adheres to the Consolidated Standards of Reporting Trials (CONSORT) guidelines for reporting randomized clinical trials^9^. The trial was designed in accordance with the Declaration of Helsinki^10^. This trial was conducted in compliance with the original protocol. The full protocol of the study is available by request to the corresponding author.

This trial was a randomized, double-blinded, controlled trial. Eligible patients were those scheduled for elective bladder or prostatic transurethral resection under general anesthesia from August 2018 to October 2018 at the Hospital Universitario de Gran Canaria Dr. Negrín, Las Palmas de Gran Canaria, Spain. We excluded patients with current infections, those taking antipyretics within 24 h before surgery, patients with neuropathy, thyroid disorders, marked peripheral vascular diseases, skin lesions, or history of hypersensitivity to skin contact devices. Patients expected to have a transurethral resection lasting longer than one hour, and those who declined consent were also excluded.

### Intervention

Prewarming was performed in the pre-anesthesia room using a forced-air blanket (WarmTouch total body blanket, Covidien, Mansfield, USA) covering the entire body. Temperature output of the warmer (WarmTouch Model WT-5900, Covidien) was set at maximum level (43.0 °C).

### Outcomes

For assessing hypothermia, we selected as the primary outcome the difference in core temperature among treatment groups from the time of arrival to the pre-anesthesia room to the end of surgery. Secondary outcomes included the incidence of postoperative hypothermia, shivering, pain intensity 30 min upon arrival at the PACU, length of PACU stay, and postoperative complications.

### Sample size

We estimated the sample size based on data from our previous observational study in 56 patients receiving spinal anesthesia, in which hypothermia developed in 96% of non-prewarmed patients^11^. We performed a power analysis to detect a temperature difference of 0.15 °C (± 0.05 °C) at the end of surgery. To detect differences in the esophageal temperature, 40 patients in each group were estimated to provide 80% power at an alpha level of 0.05. Surgery lasted less than 60 min and several temperature measurements were recorded intraoperatively (at 15, 30, and 45 min). Since in some patients the surgical procedure lasted less than 30 min, temperature at 30 or 45 min could not be measured. Under the expectation of missing data at 30 or 45 min in many patients, the sample size was increased to at least 293 patients. No interim analysis was performed.

### Enrolment, randomization, and masking

Eligible patients were approached the day before surgery after ensuring they met inclusion criteria and none of exclusion criteria. Subsequently, informed consent was obtained. Randomization was performed by an assistant (not involved in enrollment or data collection) using a computer-generated randomization approach with a 1:1 ratio without blocks or stratification to one of the following four groups: three prewarming groups (15, 30, or 45 min) and a control group (non-prewarming group). The computer-generated allocation sequence was done by a statistician not involved in the rest of the trial. The assistant was the only person with access to documents indicating the randomization group in which each patient was allocated. After randomizing each patient, the assistant reported the duration of prewarming by a phone call to the nurse-in-charge of patient care in the pre-anesthesia room. Patients were unaware of the duration of prewarming. In patients allocated to the control group, the same blanket was placed over the body without connecting it to the warmer. The attending intraoperative anesthesiologist, the anesthesiologist in-charge of care in the PACU, and the assistant reviewing postoperative data were blinded to patient’s allocation.

### Procedures

Before starting the trial, nurses in-charge of temperature monitoring in the pre-anesthesia room were trained for correct measurements using tympanic thermometers. An otoscope was used in each patient to ensure that tympanic membrane could be visualized. After checking that the probe tip was clean, a cover was placed. Then, the probe tip was inserted into the ear canal without an ear tug and seated in the ear canal by rotating the handle a quarter turn toward the jaw. To reduce intra-observer variability in temperature measurements, we selected the mean value of three consecutive measurements in each patient^11^. Hypothermia was defined as a body temperature < 36.0 °C.

#### Preoperative phase

Patient’s age, gender, body weight and height, American Society of Anesthesiologists (ASA) physical status, and surgical indication of transurethral resection (prostatic or bladder) were recorded. Upon arrival at the pre-anesthesia room, temperature was measured using a tympanic thermometer (Genius 2 Tympanic Thermometer and Base, Covidien, Mansfield, USA). Patients were prewarmed as established in randomization sequence. To avoid delays between the intervention and the induction of anesthesia, prewarming started before the end of surgery in the previous patient, and induction of anesthesia of prewarmed patient was performed once prewarming time finished. After prewarming and before transferring the patient to the operating room, temperature was measured again.

#### Intraoperative phase

Patients were actively warmed intraoperatively using a forced-air body blanket (WarmTouch upper body blanket, Covidien) positioned over the upper part of the body. Patients were pre-medicated with intravenous midazolam (1–3 mg) at the discretion of the attending anesthesiologist. Intraoperative monitoring included non-invasive arterial pressure, heart rate, electrocardiography, peripheral arterial oxygen saturation, and bispectral index (BIS, Covidien). General anesthesia was performed using fentanyl (1–2 mcg kg^−1^) and propofol with effect-site target-controlled infusion (TCI, B.Braun, Melsungen, Germany) to maintain BIS 40–60. A laryngeal mask (Ambu AuraGain, Ballerup, Denmark) was used to provide mechanical ventilation. After anesthetic induction, temperature was measured using an esophageal thermometer (Mon-a-Therm, Covidien) placed through the drainage tube of the laryngeal mask, and recorded at 15-min intervals from anesthesia induction to the end of surgery. Neither intravascular fluids nor bladder irrigating fluids were warmed. Room temperature, volume of intravenous fluids, and volume of infused glycine were also recorded.

#### Postoperative phase

Patients were transferred to the PACU after surgery, where an independent clinician was in-charge of care. The presence of shivering upon arrival (as defined as been visible by an observer), pain intensity at 30 min after arrival at PACU (using a numerical rating scale, NRS), and length of PACU stay were recorded. Patients were transferred to the hospital ward once the Aldrete modified scale^12^ was higher than 8. During the postoperative hospital stay, a trial investigator recorded the presence of cardiovascular complications (cardiac arrest, myocardial infarction, unstable angina, arrhythmias), as assessed by an independent clinician (when a patient had cardiovascular symptoms, appropriate complementary tests were performed). Postoperative analgesic requirements were controlled by the clinician responsible for managing the patient (not involved in the trial). These analgesic requirements were not recorded for the study, since due to the characteristics of the surgeries, it was assumed that it would consist only of non-opioid analgesics. The need for re-operation due to severe postoperative bleeding, and blood transfusion requirements (defined by the need of ≥ 1 red blood cell package) were also recorded. Due to the characteristics of the surgery, surgical wound infection was not recorded.

### Statistical analysis

Categorical variables are expressed as frequency and percentages and continuous as mean and standard deviation (SD) when data followed a normal distribution, or as median and interquartile range (IQR 25th–75th percentiles) when distribution departed from normality. Percentages were compared using the Chi-square test, means by the t-test, and distributions by the Kruskal–Wallis test for independent data.

To assess the effects of prewarming on temperature evolution throughout the surgery, we fitted a repeated measure multivariate analysis of covariance (MANCOVA) model to the temperature response vector (On arrival at the pre-anesthesia room, Before entering the operating room, 15 min after induction, 30 min after induction, 45 min after induction, At the end of surgery), using the prewarming time as the categorical main effect. The effects of each time-periods of prewarming and the prewarming-time interaction were contrasted using the corresponding F-tests. The model framework allowed us to estimate the marginal means of temperature according time and prophylaxis type directly from parameters of the fitted model. Mean temperatures throughout the surgery according to each treatment group were estimated using 95% confidence intervals and multiple comparisons of these means were made between the control group and each prewarmed group using the test of Dunnet.

Length of stay in PACU among groups was analyzed using one-way ANOVA for independent samples. For each prewarmed group, the survival functions corresponding to the length of stay in the PACU were estimated using the Kaplan–Meier method. Comparisons between groups were made using the log rank test.

Statistical significance was set at p < 0.05. Data were analyzed using the R Core Team (2016), version 3.3.1 (R: A language and environment for statistical computing. R Foundation for Statistical Computing, Vienna, Austria. URL: https://www.R-project.org/).

## Results

**Figure 1:**
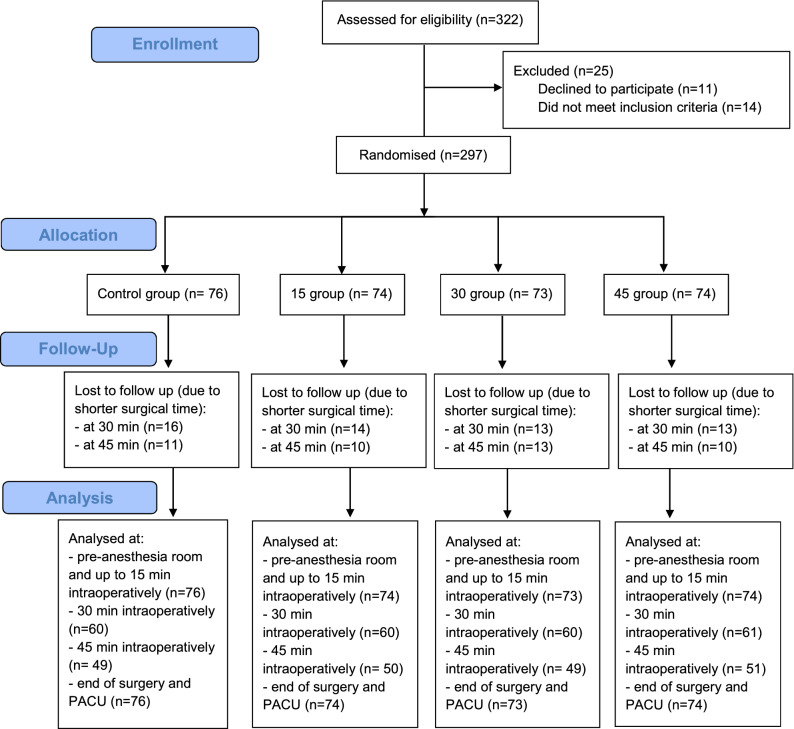
Flow chart diagram.

Between August and October 2018, we enrolled 297 patients and randomly assigned 76 patients to the control group, 74 patients to the 15-min prewarming group, 73 patients to the 30-min prewarming group, and 74 patients to the 45-min prewarming group (Fig. 1). Patient characteristics, temperature of the operating room, intravenous volume infused, duration of surgery, and amount of glycine instilled were similar in all groups (Tables 1 and 2). In the pre-anesthesia room, 16.5% (49/297) of patients had a temperature lower than 36 °C, while the rest of patients were normothermic. We did not find differences among groups regarding the core body temperature upon arrival at the pre-anesthesia room (Table 3).

**Table 1:** Characteristics of patient preoperative variables.

	Control (n = 76)	15-min (n = 74)	30-min (n = 73)	45-min (n = 74)
Age, years	70 ± 13	73 ± 11	70 ± 12	71 ± 11
Male gender, No. (%)	66 (87)	64 (86)	49 (67)	67 (91)
Body mass index, kg m^−2^	27.5 ± 4.6	27.7 ± 4.1	27.5 ± 4.3	28.0 ± 4.5
Body surface area, m^2^	1.9 ± 0.2	1.9 ± 0.2	1.9 ± 0.2	2.0 ± 0.2
**Type of transurethral resection, No. (%)**				
Bladder	61 (80)	67 (90)	63 (86)	56 (76)
Prostatic	15 (20)	7 (10)	10 (14)	18 (24)
**ASA physical status, No. (%)**				
I	3 (4)	0 (0)	3 (4)	1 (1)
II	27 (35)	32 (43)	31 (43)	30 (41)
III	44 (58)	38 (51)	38 (52)	41 (55)
IV	2 (3)	4 (5)	1 (1)	2 (3)

Data are expressed as mean ± SD or frequency and percentage. ASA, American Society of Anesthesiologists; 15-min, prewarmed for 15 min; 30-min, prewarmed for 30 min; 45-min, prewarmed for 45 min.

Body Surface Area equation: Square root of (Weight (kg) × height (cm)/3600).

**Table 2:** Characteristics of intraoperative variables.

	Control (n = 76)	15-min (n = 74)	30-min (n = 73)	45-min (n = 74)	*P* Value
Duration of surgery (min)	30 [20–45]	20 [15–35]	25 [20–40]	25 [15–39]	0.209
Operating room temperature (°C)	23.0 [22.6–23.1]	22.9 [22.5–23.1]	22.8 [22.3–23.1]	23.0 [22.7–23.2]	0.260
Fluid therapy (ml)	300 [200–500]	300 [200–400]	300 [200–500]	300 [200–500]	0.116
Glycine (l)	5 [3–10]	4 [3–7]	5 [3–9]	3 [3–10]	0.460

Data are expressed as median [IQR]. 15-min: prewarmed for 15 min. 30-min: prewarmed for 30 min. 45-min: prewarmed for 45 min.

**Table 3:** Perioperative temperature in each group (°C) estimated from the repeated measures model.

Time	Control (n = 76)	15-min (n = 74)	30-min (n = 73)	45-min (n = 74)
On arrival at the pre-anesthesia room	36.6 [36.4; 36.8]	36.5 [36.3;36.7]	36.5 [36.3; 36.7]	36.5 [36.3; 36.7]
Before entering the operating room	36.5 [36.3; 36.7]	36.7 [36.5; 36.9]	36.8 [36.7; 37.0]	36.9 [36.7; 37.1]
15 min after induction	36.4 [36.2; 36.6]	36.6 [36.4; 36.9]	36.7 [36.5; 36.9]	36.8 [36.6; 36.9]
30 min after induction	36.1 [35.9; 36.2]	36.4 [36.2; 36.6]	36.5 [36.3; 36.7]	36.6 [36.4; 36.8]
45 min after induction	35.8 [35.6; 36.0]	36.2 [36.0; 36.4]	36.4 [36.2; 36.6]	36.4 [36.2; 36.6]
At the end of surgery	35.5 [35.2; 35.7]	36.1 [35.9; 36.4]	36.2 [35.9; 36.4]	36.3 [36.1; 36.5]

Data are expressed as marginal means [95% CI]. 15-min: prewarmed for 15 min; 30-min: prewarmed for 30 min; 45-min: prewarmed for 45 min.

Mean body temperature of control group before entering into the operating room was 36.5 ± 0.5 °C. After prewarming, body temperature was higher in two prewarmed groups (between 15-min—control difference 0.3 °C, 95% CI 0.1–0.4, p = 0.004; between 30-min—control difference 0.2 °C, 95% CI 0.0–0.3, p = 0.041). Between 45-min—control difference was 0.1 °C (95% CI 0.1–0.3, p = 0.203). Temperature in the control group at the end of surgery was 35.8 ± 0.6 °C. At the end of surgery, temperature was higher in prewarmed groups than in the control group (between 15-min—control group difference 0.5 °C, 95% CI 0.3–0.7, p < 0.001; between 30-min—control group difference 0.5 °C, 95% CI 0.3–0.7, p < 0.001; and between 45-min—control group difference 0.5 °C, 95% CI 0.3–0.7, p < 0.001). No differences were found in intraoperative temperature among prewarmed groups.

**Figure 2:**
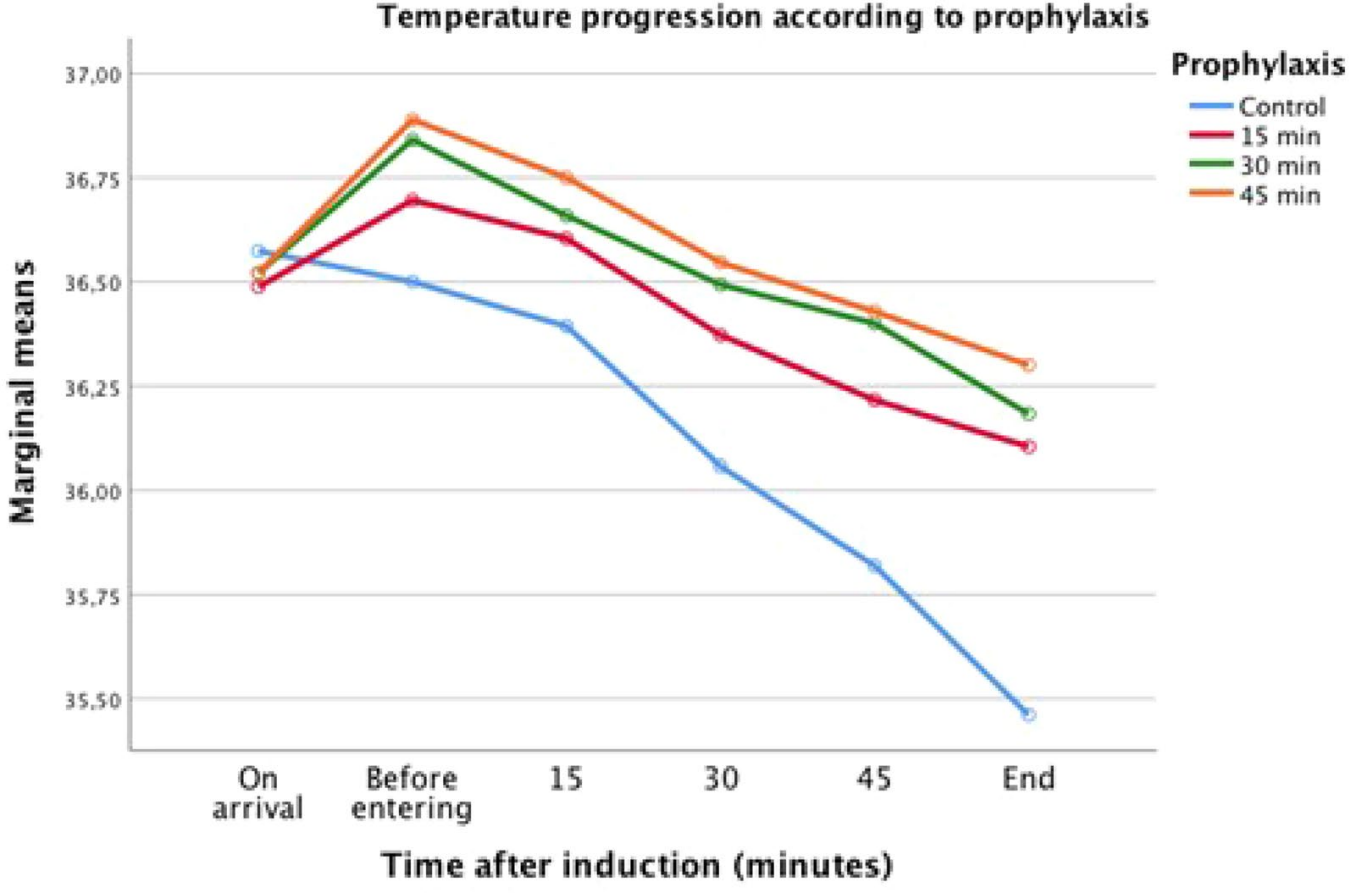
Repeated measures model for perioperative temperatures according to the prewarming duration: estimated marginal means (°C). 15-min: prewarmed for 15 min; 30-min: prewarmed for 30 min; 45-min: prewarmed for 45 min. The effects on temperature of the prewarming for 15 min did not show a statistically significant difference with the control group (p = 0.099). However, the effects showed statistically significant differences with the groups prewarmed for 30 min (p = 0.010) and for 45 min (p = 0.003).

The repeated measures multivariate model shows that the effects of duration of prewarming were statistically significant (p < 0.001) and also, the effects of interaction time-treatment (p < 0.001). Table 3 show the marginal means of perioperative temperatures adjusted by the model according the duration of prewarming and time elapsed after induction. Figure 2 shows the trajectories of the temperatures throughout the surgery in each group. Prewarming for 30 min maintained mean temperature throughout the surgery 0.4 °C (95% CI 0.1–0.7) above the control group (p = 0.010) and prewarming for 45 min maintained mean temperature 0.4 °C (95% CI 0.1–0.7) above the control group (p = 0.003). However, when prewarming lasted only 15 min, mean perioperative temperature throughout the surgery did not reach a significant difference when compared to the control group (p = 0.099) (Table 4).

**Table 4:** Comparison of mean temperatures throughout surgery between each prewarmed group with the control using the Dunnett test.

Prewarming	Difference of temperature means [95% CI]*	P-value
**Control**		
15 min	0.3 [− 0.0; 0.6]	0.099
30 min	0.4 [0.1; 0.7]	0.010
45 min	0.4 [0.1; 0.7]	0.003

(*) Differences are between the control group minus each prewarmed group.

**Figure 3:**
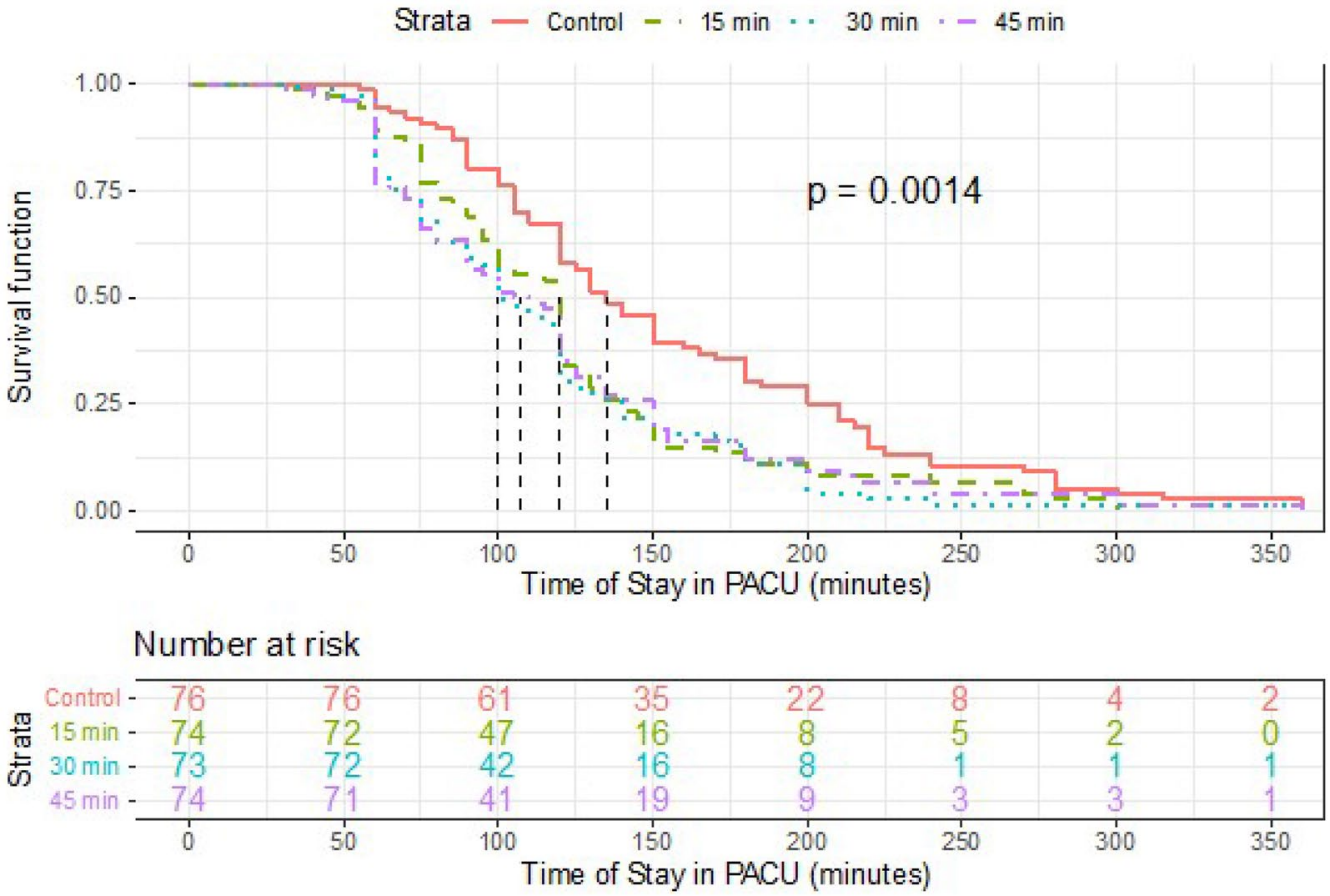
Survival function for the length of stay in PACU according to the prewarming duration. The median length of stay obtained in each group were as follows: in the control group, 135 min; in the group prewarmed for 15 min, 120 min; in the group prewarmed for 30 min, 100 min; and in the group prewarmed for 45 min, 108 min.

At the end of surgery, 54% (41/76) of patients in the control group, 23% (17/74) in the 15-min group, 25% (18/73) in the 30-min group, and 30% (22/74) in the 45-min group developed hypothermia (p < 0.001). Upon admission to PACU, 42% (32/76) of patients in the control group had shivering episodes. No shivering was observed in the 30-min group, and it only affected 4% (3/74) of patients in the 15-min and the 45-min groups (OR 0.058, 95% CI 0.017–0.201). Prewarmed groups also showed a lower NRS (between-group difference for 15-min—control 1.0, 95% CI 0.8–1.2, p < 0.001; between-group difference for 30-min—control 1.2, 95% CI 0.9–1.4, p < 0.001; between-group difference for 45-min—control 1.1, 95% CI 0.9–1.3, p < 0.001) and a shorter length of stay in PACU (between-group difference for 15-min—control 34 min, 95% CI 13–55, p = 0.002; between-group difference for 30-min—control 42 min, 95% CI 21–63, p < 0.001; between-group difference for 45-min—control 38 min, 95% CI 16–60, p = 0.001) (Table 5). The length of stay in the PACU showed statistically significant differences among groups (p = 0.001). However, there were no significant differences between the three prewarmed groups (p = 0.800). Therefore, in order to reduce the time that the patient spends in the PACU, any prewarming duration (15, 30 and 45 min) is superior to the control (Fig. 3).

**Table 5:** Presence of shivering, pain (NRS) and length of stay in PACU in each group.

	Control (n = 76)	15-min (n = 74)	30-min (n = 73)	45-min (n = 74)	P Value
Presence of shivering, No. (%)	32 (42)	3 (4)	0 (0)	3 (4)	< 0.001
**NRS, No. (%)**					
1	8 (11)	49 (66)	55 (75)	52 (70)	< 0.001
2	33 (43)	17 (23)	17 (23)	18 (24)	
3 / 4	35 (46)	8 (11)	1 (1)	4 (5)	
Length of stay in PACU (min)	156 ± 72	122 ± 58	114 ± 56	118 ± 64	0.001

PACU: Post-Anesthetic Care Unit; NRS: numerical rating scale; 15-min: prewarmed for 15 min; 30-min: prewarmed for 30 min; 45-min: prewarmed for 45 min.

Data are expressed as percentage and frequency or mean ± SD.

In the control group, two patients suffered from post-surgical cardiac complications (atrial fibrillation and thoracic pain) and one patient needed re-operation due to post-surgical bleeding. No patients in prewarmed groups suffered from cardiac complications or needed re-operation. These differences were not statistically significant (p = 0.119 and p = 0.4040, respectively). Of the two patients developing cardiac complications, only one patient had postoperative shivering. One patient in the 15-min group and two patients in the 45-min group needed perioperative blood transfusions. In control group and in the 30-min group, no patients needed postoperative blood transfusions, but this difference was not statistically significant (p = 0.293). No complications due to prewarming were observed.

## Discussion

In this randomized, double-blind, controlled trial, the application of short-time periods of active prewarming before bladder or prostatic transurethral resection reduced the prevalence of redistribution hypothermia. Prewarming also reduced postoperative shivering, postoperative pain intensity, and length of stay in the PACU.

Perioperative hypothermia affects up to 20–50% of patients undergoing general anesthesia^13^. Once the temperature has decreased, treatment of redistribution hypothermia is difficult, since the application of heat to the superficial compartment takes a long time to reach the core compartment. There are two reasons why redistribution hypothermia occurs. First, general anesthetic agents impair autonomic thermoregulatory control, decreasing the vasoconstriction threshold 2–4 °C. The second major factor is the degree of temperature gradient between the core and peripheral compartments^4^. Peripheral compartments (legs and arms) represents approximately 48% of the total body mass, and redistribution hypothermia contributes 81% to the temperature drop, corresponding to a redistribution of 46 kcal during the first hour after general anesthesia induction^14^. The application of warming only during the intraoperative period would not be sufficient to compensate this problem. The prevention of perioperative hypothermia by intraoperative warming using forced air devices seems to be more effective when a greater body surface area of the patient is covered^15^. Forced-air warming increases peripheral heat content (69 kcal) during the first 30 min of warming^16^. During the lithotomy position necessary to perform transurethral resection, large areas of patient's surface are uncovered. Therefore, intraoperative warming alone is insufficient to maintain normothermia in this type of surgery, and prewarming could diminish the redistribution from the core to the peripheral tissues. In our study, short-time periods of active prewarming reduced redistribution hypothermia by 0.5 °C and reduced the prevalence of hypothermia at the end of surgery by 24–31%.

We observed a lower prevalence of preoperative hypothermia than expected^11,17^. The main factors related to perioperative temperature decrease include the preoperative hypothermia^18,19^, or the duration of surgery^20^. Although most of these risk factors are not modifiable, there are some modifiable factors, such as prewarming. Active prewarming using forced air devices does not increase core body temperature but reduces core-to-peripheral redistribution of heat^21^. It was initially established that prewarming for 60 min was required to prevent intraoperative hypothermia^21–25^. In other studies, prewarming for 60 min did not prevent the occurrence of hypothermia, but it attenuated redistribution hypothermia^26^. In patients under general anesthesia, 30 min of prewarming appeared to reduce hypothermia^16,27,28^, and even 10–15 min reduced temperature decrease and postoperative shivering^29–31^. However, these studies were performed on surgeries lasting more than 60 min. Studies conducted on surgeries shorter than 60 min also showed that prewarming decreased the prevalence of perioperative hypothermia^32–34^, but results cannot be extrapolated to our population because they were obtained from pregnant women with the inherent physiological implications. In a previous study performed on men submitted to transurethral resection under spinal anesthesia, short prewarming (15–30 min) reduced the prevalence of hypothermia at the end of the procedure^11^.

In our trial, prewarming for 45 min also succeeded in reducing the occurrence of hypothermia at the end of surgery. Our results are consistent with a meta-analysis of previous prewarming trials^35^. We also observed that prewarming for less than 45 min reduced the prevalence of postoperative shivering, the intensity of postoperative pain, and the length of stay in the PACU. Postoperative shivering is one of the most important causes of patient’s discomfort and prewarming can increase patient comfort^11,35^. Most post-anesthetic shivering-like tremors are presumably thermoregulatory responses to hypothermia. Nevertheless, shivering can appear in normothermic patients, triggered by the presence of pain^36^. Therefore, patients in the control group might present more episodes of shivering, not only because they were hypothermic, but also because they had more pain. Despite that transurethral resection is a type of surgery associated with mild pain, we found differences in postoperative pain among groups. It could be argued that suffering greater discomfort leads to more complaints of pain. However, temperature and pain signals travel together through the same nerve fibers, synapsing in the dorsal horn of the spinal cord. Thermoregulatory response to the increase in temperature could inhibit the response to pain. There are few studies evaluating the relationship between hypothermia and pain in the postoperative period with contradictory results^29,37–39^.

We acknowledge some limitations of our study. First, the gold standard for central temperature monitoring is via a catheter in the pulmonary artery^4,40^. Esophageal thermometers are less invasive, but it is not feasible to place them prior to induction. In the pre-anesthesia room, tympanic temperature measurement is easily accessible and comfortable, with a reported accuracy of ± 0.1°C^40^. We could have obviated data collection of preoperative temperature, but this information allows us to know the preoperative core temperature and how prewarming modifies it. Therefore, since comparison among groups at each time-period was performed using the same thermometer, we believe that our results are valid. Second, we analyzed each time-point without considering temperature progression in each patient. Third, the study was performed on transurethral resection, where bladder irrigation with glycine in the operating room temperature is used. Bladder irrigation produces trivial cooling^41^, and is therefore unlikely to have influenced our conclusions.

## Conclusions

This randomized controlled trial on surgeries lasting less than 60 min under general anesthesia shows that active prewarming for at least 15 min reduces redistribution hypothermia, the decrease of body temperature throughout perioperative period, and the prevalence of hypothermia at the end of surgery. However, at the end of the surgery, each of the prewarming time-periods (15, 30 or 45 min) are comparably effective, and about 0.5 °C higher than no prewarming. One of our most clinically relevant findings is that these prewarming time-periods also reduced postoperative shivering, postoperative pain intensity, and length of stay in PACU.
